# Schools and Measles

**Published:** 1900-04-14

**Authors:** 


### SCHOOLS AND MEASLES.

The question of closing schools during the progress of epidemics has often been discussed, and with the general result of showing that when an outbreak of disease has once fairly established itself in a town such a proceeding is of but small effect, shutting the door as it does to only one of many ways by which infection can spread from case to case. In a country district, of course, the matter is very different, and it may perhaps be broadly laid down that the desirability of closing schools, and especially of infant schools, depends principally upon the extent to which such schools are the principal, or perhaps the sole, means of intercommunication between the susceptible children of the district. At a recent meeting of the Epidemiological Society, Mr. Robinson, M.O.H. to the county of Lancaster, read a paper on the distribution and control of epidemic measles, based on an experience of over two thousand cases during an epidemic at Burton-on-Trent in 1898, the estimated population of the town at that time being 51,664. Among the general conclusions which he drew from his observations were, that measles introduced into a district through infant school, Sunday school, or day school, spread with rapidity, and to an extent inversely proportionate to the previous frequency of epidemics in the districts; in other words, in proportion to the number of susceptible children in the district, unless early and prompt closure of the school were enforced; that when introduced into a district by means of isolated cases it did not spread to any extent until the infant schools became infected; that Sunday schools played a prominent part in spreading the disease; that in the great majority of cases "babies" were not attacked until after the invasion of the schools, showing that the disease was in many cases taken home, and lastly, as is only to be expected, that house to house infection became a danger to babies in proportion to the proximity of the infected house. As to obtaining control over the disease, he considered that closure, as hitherto practised, was nearly always enforced too late; that if carried out before the infant schools had become infected great benefit would accrue, not only to the infants, but also to the babies, and that when it became necessary to close the infant schools the sanitary authorities should be empowered to close the Sunday schools also. Attention may be drawn to an interesting observation made by Mr. Robinson, namely, that where outbreaks had recurred biennially, as tends to happen when the disease is left to itself, the incidence was almost confined to the infant department, while in those cases in which there had been no epidemic for six or more years the boys and girls were also attacked in proportions of from 10 to 20 per cent. It is then open to those who object to control to say that the effect of these measures is but to push off the evil day, and, instead of letting the children get over the disease during their infancy, to leave us with an adult population far more susceptible to measles than is the case at the present day. No doubt there is something in this contention. On the other hand, measles is a disease which is probably far more fatal to infants than to those of greater age, while it seems extremely likely (although English statistics are of no great value on this point) that the incidence of measles among the adult population, even though not protected by previous attack, would not be so great as it is among those of tender years. Nevertheless, it is to be admitted that we are as yet without any very accurate knowledge as to what would be the effect of leaving a large proportion of the adult population in an unprotected condition in regard to measles, a point which has a somewhat important bearing upon the discussion.

